# Supplementary material for: Splice-Junction-Based Mapping of Alternative Isoforms in the Human Proteome
Source: Cell Rep. Author manuscript; Available in PMC 2020 Jan 15. (PMC6961840; doi:10.1016/j.celrep.2019.11.026)
Supplement: 2 [file NIHMS1546469-supplement-2.zip › DF1/PXD010154/Ovary/Ovary_8_PPP1R12A_ISEMEEELKR.pdf]

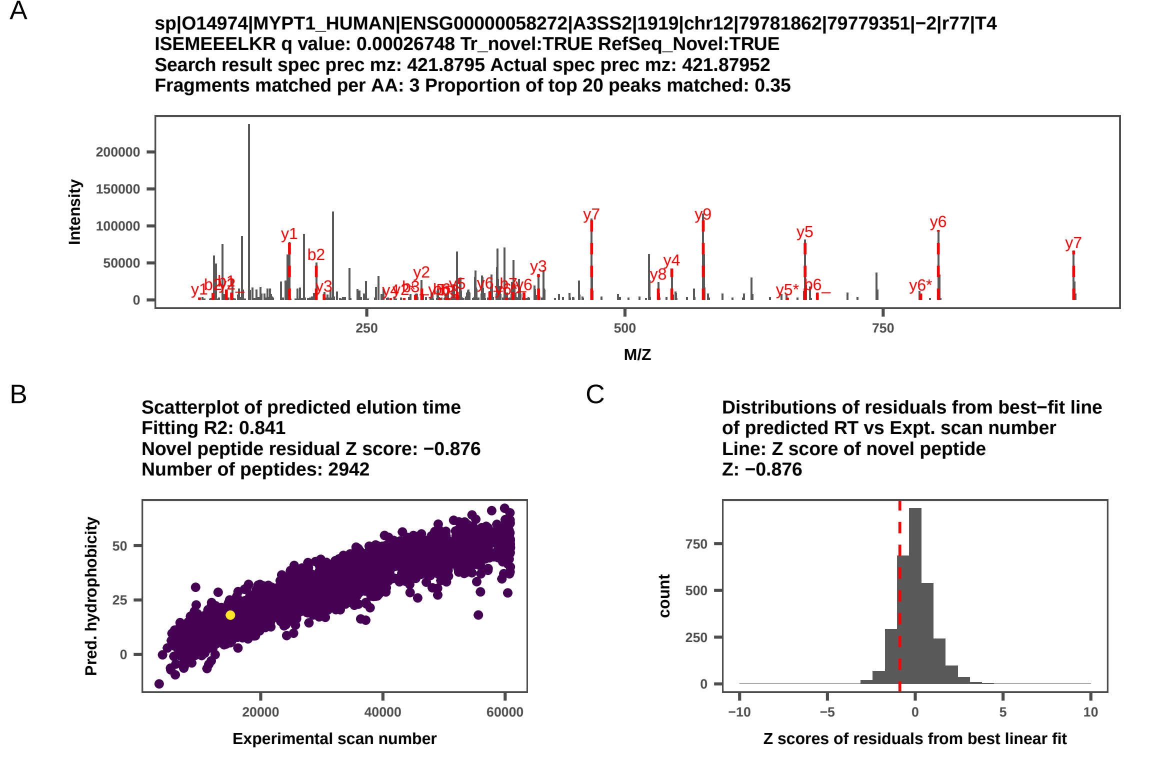
A
sp|O14974|MYPT1_HUMAN|ENSG00000058272|A3SS2|1919|chr12|79781862|79779351|−2|r77|T4
ISEMEEELKR q value: 0.00026748 Tr_novel:TRUE RefSeq_Novel:TRUE
Search result spec prec mz: 421.8795 Actual spec prec mz: 421.87952
Fragments matched per AA: 3 Proportion of top 20 peaks matched: 0.35
Intensity
200000
150000
100000
50000
0
y1
b2
y3
y2
y3
y7
y8
y4
y9
y5*
y5
b6
y6*
y6
y7
250
500
750
M/Z
B
Scatterplot of predicted elution time
Fitting R2: 0.841
Novel peptide residual Z score: −0.876
Number of peptides: 2942
Pred. hydrophobicity
50
25
0
20000
40000
60000
Experimental scan number
C
Distributions of residuals from best−fit line
of predicted RT vs Expt. scan number
Line: Z score of novel peptide
Z: −0.876
count
750
500
250
0
−10
−5
0
5
10
Z scores of residuals from best linear fit
